# Implementing a Social Presence-Based Teaching Strategy in Online Lecture Learning

**DOI:** 10.3390/ejihpe14090170

**Published:** 2024-09-21

**Authors:** Liangliang Xia, Lianghui Wang, Changqin Huang

**Affiliations:** 1 School of Education Technology, Beijing Normal University, Beijing 102206, China; xialiangliang@mail.bnu.edu.cn; 2 Key Laboratory of Intelligent Education Technology and Application of Zhejiang Province, Zhejiang Normal University, Jinhua 321004, China

**Keywords:** social presence, online interaction, academic performance, social network

## Abstract

Previous studies have focused on the design of video lectures to improve students’ social presence by enhancing instructor presence for learners in lecture-based online courses; however, there has been limited emphasis on the peer presence in which learning from video lectures takes place. This study’s first objective is to develop a social presence (SP)-based teaching strategy to design online learning activities aimed at improving students’ social presence by providing social clues about peer presence and encouraging peer communication. The second objective is to compare students’ social presence, social interaction, and academic performance from lecture-based online learning supported by either a conventional teaching strategy or an SP-based teaching strategy. Using a quasi-experiment, we selected 81 Chinese university students to participate in a ten-week online course. The participants were randomly assigned to either an experimental group (EG) (*N* = 43) or a control group (CG) (*N* = 38). This study revealed that the SP-based strategy enhanced EG members’ social presence in online learning and that EG members achieved better academic performance than CG members. A significant correlation was found between the EG members’ academic performance and their social presence. The researchers also identified more concentrated social network sociograms with more cohesive subgroups in the EG members’ online interactions. The results indicate the necessity of applying an SP-based teaching strategy in lecture-based online courses to promote students’ social presence, social interaction, and academic performance.

## 1. Introduction

Within higher education, online teaching with the support of information and communication technology has been largely recognized as an important method by which to implement instruction [1]. However, despite the great potential of technology in online education, not all students can achieve success in online education with the support of technology, given the technological gap and the limited technological proficiency among both teachers and students [2,3]. In the current online education environment, lecture-based teaching methods have become increasingly popular, especially in large-scale online open courses and during emergency situations such as that of COVID-19 [4,5,6,7,8]. However, this teaching practice may result in students’ online isolation and could be seen as lacking the ability to create learning-conducive interactions, which in turn could impede students from achieving their learning objectives through online classes [9,10,11,12].

Researchers have found that social presence, as a key construct in online learning, plays a critical role in student online course engagement, emotional experience, and online interaction [13,14,15,16,17,18]. In addition, social presence has positive effects on students’ learning outcomes [19,20,21]. The community of inquiry (COI) framework assumes that effective online learning occurs when students experience sufficient social presence, teaching presence, and cognitive presence while learning online [16]. The primary role of social presence in the COI framework is to create group cohesion and an open communication environment associated with higher-order learning [22]. Given the impact of social presence on students’ online learning, the practice of transferring lecture-based instruction online can be improved by enhancing students’ social presence.

Online educators have continued to explore various methods to establish and sustain social presence in online courses [23]. Previous research has focused primarily on using video design to enhance students’ social presence in lecture-based online courses, including factors such as the instructor’s eye gaze, the instructor’s image in accompanying lecture slides, the instructor’s gaze guidance, and interactive visualization tools [8,24,25,26]. However, while much attention has been given to the design of video lectures to provide more instructor presence for learners, there has been limited emphasis on the social environment in which learning from video lectures takes place [7]. Social psychology theory emphasizes the role of another’s presence in learning [27]. Another presence in lecture-based online learning includes not only the instructor presence but also the co-learner presence. For example, Brand et al. (2003) [28] discovered that, compared with learners who learned alone, those who learned in the presence of peers who were participating in the same activity were better at solving learning tasks. Therefore, in lecture-based learning, designing activities for students with peers can help improve students’ academic performance and social presence.

The present study aimed to explore whether a social presence (SP)-based teaching strategy can provide students with important social cues about the presence of peers in a lecture-based online learning community, thereby enhancing students’ social presence and resulting in meaningful learning. Previous research has not directly designed instructional strategies that address the presence of peers based on theoretical frameworks in the field of social presence. The theoretical framework of social presence describes the dimensions of social presence, and an in-depth understanding of social presence helps to scientifically design teaching strategies to enhance the presence of peers, thereby enhancing students’ perceived social presence. Therefore, based on Biocca et al.’s (2003) [29] social presence theory, this study proposed an SP-based teaching strategy to improve lecture-based online instruction by designing online activities that aimed to enhance students’ social presence. Additionally, the researchers implemented six online learning activities under the guidance of this SP-based teaching strategy as an intervention that focused on improving students’ social presence in an online course. To examine the effect of the SP-based teaching strategy, this study compared students’ social presence, social interaction, and academic performance from lecture-based online learning supported by either a conventional teaching strategy or an SP-based teaching strategy. The research questions were as follows:

(1) Will the SP-based teaching strategy significantly enhance students’ social presence in online learning?
(2) Will the SP-based teaching strategy enhance students’ online interaction?
(3) Will the SP-based teaching strategy significantly improve students’ academic performance at the end of the online course?

## 2. Literature Review

### 2.1. Lecture-Based Instruction in Online Courses

Lecture-based instruction is the teaching method that most teachers use in their regular online teaching. Teachers usually deliver online lecture-based instruction through synchronous interaction (e.g., providing live online lectures via Zoom and Ding Talk) and asynchronous interaction (e.g., providing prerecorded lectures and discussion forums via Moodle) [30]. Synchronous instruction allows real-time communication between teachers and students despite their geographical distance [31,32]. Asynchronous instruction provides greater flexibility to suit students’ schedules and allows students to review video lectures repeatedly at their convenience [33]. Thus far, the findings regarding the effects of synchronous and asynchronous instruction have been mixed, as researchers have found that both synchronous and asynchronous instruction have advantages and limitations. In terms of students’ perceptions, some researchers have found that students prefer instruction in an asynchronous format [34,35]. In contrast, Moridani (2007) [36] discovered that students had lower satisfaction with asynchronous lectures and preferred live interactive sessions. For students’ learning performance, Kubey et al. (2010) [37] reported that students receiving synchronous online instruction had lower academic achievement than those receiving asynchronous online instruction. However, Somenarain et al.’s (2010) [38] research showed no significant difference between synchronous online instruction and asynchronous online instruction in terms of students’ course outcomes.

It has become widely accepted that online learning requires more interaction to maintain students’ course engagement and retention [39,40,41,42]. However, lecture-based online instruction, as a teacher-centered teaching approach, may cause several issues, such as a lack of social and emotional support during teacher–student and peer interactions, neglect of students’ diverse learning needs and preferences, and monotonous online learning activities [20,43,44,45,46,47]. The above issues hinder students from achieving their learning goals in online courses. Therefore, teachers who rely entirely on lecture-based instruction in their online courses need to apply effective teaching strategies to improve their students’ social presence, online interaction, and academic performance.

### 2.2. Social Presence

Short et al. (1976) [48] defined social presence as “the degree of salience of the other person in the interaction” in the field of social psychology and communication. Short’s team focused on the degree of technology users’ awareness of other people and conceptualized social presence as a quality of communication media. That is, some media were considered to have a greater capability to convey social presence (e.g., video-based conferencing), while others, such as text-based communication, had a weaker capability to convey social presence. Researchers subsequently questioned whether media quality determined social presence and reconceptualized social presence as a feeling of having someone socially present in one’s life even if they were not physically in the same space [49]. For instance, Rourke et al. (1999) [50] defined social presence as “the ability of learners to project themselves socially and affectively into a community of inquiry.” In 1999, Garrison et al. [51] introduced the CoI model, emphasizing the crucial role of social presence in supporting cognitive presence. They proposed that social presence indirectly facilitated the process of critical thinking carried out by the community of learners. In this study, we describe social presence as the ability to perceive others in an online environment.

#### 2.2.1. Biocca et al.’s Social Presence Theory

The dimensions of social presence, including intimacy, immediacy [48], social respect (e.g., timely response), open mind (e.g., self-disclosure), social identity (e.g., learner’s characteristic), social sharing (e.g., sharing information) [52], interactivity, social context (e.g., social relationships), online communication [53], cohesion [51], and privacy [54], have been proposed over the past four decades. In this study, we chose Biocca et al.’s (2003) [29] dimensions of social presence because they were comprehensive and hierarchical and were thus more instructive for the present research.

Biocca et al. (2003) [29] proposed a robust theory that outlines three dimensions of social presence by reviewing existing definitions of social presence. These dimensions are co-presence, psychological involvement, and behavioral engagement. Co-presence “is grounded on the basic sensory awareness of other” [29] and “implies the reception of embodied messages” [55]. Together, these describe a feeling of being with others in the same space and with mutual awareness, even when these others are physically separate. Psychological involvement emphasizes the deeply immersed feeling of another person, such as people’s modeling of the intelligence of the other rather than just the co-presence of a body, and considers the actions of the body as cues for the activation of the body’s intellectual state [29]. Hwang and Park (2007) [56] referred to psychological involvement as emotional connectedness. In other words, psychological involvement involves people’s perception of others’ thoughts, cognition and emotions, and focuses on people’s expression of their emotions and opinions on issues and the exchange of opinions with each other through social interaction and communication. Biocca et al. (2001) [57] described behavioral engagement as “the degree to which the observer believes his or her actions are interdependent, connected to, or responsive to the other and the perceived responsiveness of the other to the observer’s action.” Specifically, behavioral engagement refers to behavioral interaction, mutual assistance, and dependent action [57].

Biocca et al.’s (2003) [29] social presence theory was mainly used to help people understand the concept of social presence [13,15,58,59]. In addition, it is an important theoretical framework for analyzing social presence and related studies. For instance, Kim et al. (2016) [13] investigated the predictive relationship between the two dimensions of social presence (i.e., co-presence and psychological involvement) and students’ online learning experiences. They found that co-presence and psychological involvement explained 50% of the total variance in teacher–student relationship satisfaction, 51% of the variance in class satisfaction, and 57% of the variance in perceived knowledge gain.

#### 2.2.2. The Role of Social Presence in Online Learning

Previous research has shown that social presence can positively influence students’ learning experiences, such as course and instructor satisfaction [17,58,60], perceived learning [13,61], learning engagement [62], learning performance [19,21], and learning motivation [18,63]. For example, Cobb and Susan (2011) [64] have demonstrated that social presence explained 44% of the total variance in overall learner satisfaction and 36% of the total variance in student perceived learning. Furthermore, social presence can effectively predict students’ online course retention and enrollment intentions [65,66].

Several studies have investigated the relationships between students’ social presence and their academic performance. Hostetter and Busch (2013) [67] used content analysis to code students’ discussion postings and found that students who were engaged in more social presence indicators during online communication achieved better performance. Similarly, Yang et al. (2016) [20] reported that students’ perceived social presence accounted for 52.6% of the total variance in perceived learning outcomes and 15.8% of the total variance in actual course results. Moreover, Samad et al. (2019) [68] proposed a model to investigate the impact of social networking sites on students’ academic performance. They found a positive relationship between students’ social wellbeing and their academic performance, and students’ social presence could significantly influence their social wellbeing.

Finally, previous studies have shown a positive correlation between students’ social presence and online interaction [13,69,70,71]. However, the findings about the influential relationships between social presence and interactions are still mixed. For instance, Horzum (2017) [70] used structural equation modeling to investigate the relationships among interaction, structure, social presence, and satisfaction and concluded that students’ social presence was positively predicted by their online interaction. Additionally, through structural equation modeling, Wei et al. (2012) [15] showed that social presence had a significant impact on interaction.

#### 2.2.3. Effective Teaching Strategies to Improve Students’ Social Presence

A number of researchers have found that the factors influencing students’ social presence in online courses include situational variables [14,72], teacher participation and student attitudes [73], cultural backgrounds [74,75], course duration [76,77], and computer-mediated communication tools [78].

In terms of improving students’ social presence, some researchers have used various computer-based emerging technological tools that facilitate interpersonal connections to establish students’ social presence, such as online forums [79], Wike [49], Twitter [80], and VoiceThread [81]. For instance, Akcaoglu and Lee (2018) [82] used Facebook groups as outside-class social spaces in two asynchronous online courses to supplement cognitive and affective learning. The researchers found that participants perceived more social presence after joining the class Facebook group. In addition, many researchers have adopted various teaching strategies to promote social presence, such as digital storytelling [83,84], the case method of instruction [85], and online protocols [86]. Lowenthal and Dunlap (2010) [83] have demonstrated that storytelling improved students’ social presence by disclosing their personal information and relating to each other’s common experiences. Moreover, Aragon (2003) [87] adopted a series of approaches to establishing social presence, including developing welcome messages, providing frequent feedback, sharing personal stories, and using humor and emoticons.

The above strategies to enhance students’ social presence were not under the guidance of specific and creditable theoretical frameworks in the field of social presence. Therefore, it is necessary to find a specific theory to guide improvements in students’ social presence. Biocca et al.’s (2003) [29] social presence theory might be a rational choice.

## 3. The Social Presence-Based Teaching Strategy

**Figure 1:**
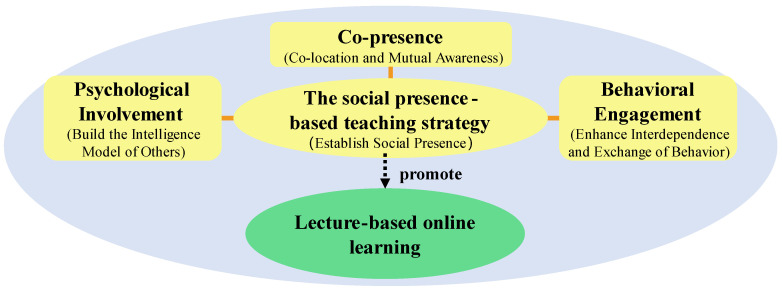
The SP-based teaching strategy.

Based on Biocca et al.’s (2003) [29] social presence theory, the present study proposed an SP-based teaching strategy that focused on improving students’ social presence in terms of co-presence, psychological involvement, and behavioral engagement in an online course (Figure 1). Furthermore, the researchers designed six activities under the guidance of the SP-based teaching strategy to establish closer connections between students and improve their social presence (Table 1).

According to Biocca et al.’s (2003) [29] social presence theory, co-presence focuses on students’ awareness of their peers’ existence, which implies that students need to receive the embodied messages of their peers. In a mediated environment, the other was frequently embodied by an avatar, agent, or simpler representational device [88]. In this study, with the support of online course platforms, all students were embodied by their personal accounts. Therefore, to improve student co-presence, students are required to participate in learner identification activities by providing personal information on the course platform (e.g., name, grade, age, specialty, and hobby) to provide individual clues that might help them sense that they were learning in an online community.

Biocca et al. (2013) [29] considered the inert bodies in virtual environments as representations that were not “inhabited” by intelligence, human or artificial, and were without spirit or agency within them. Psychological involvement hinges more on students’ models of their peers’ intelligence rather than just the co-presence of a body (Biocca et al., 2003) [29]. Therefore, students need to display their intelligence in the online course. In the present study, to help students build intelligence models for others, the researchers required the students to (a) express their online learning needs and expectations, (b) sign learning contracts to ensure that they could follow the rules of rewards and punishments during online learning, and (c) provide emotional cues during online communication (e.g., humor and emoticons in public).

Behavioral engagement emphasizes the interdependence between students’ behavior and the exchange of behavior between the students, including the reaction to others’ behavior and the degree to which one’s behavior receives feedback from others [29]. To improve students’ behavioral engagement, the researchers in the present study encouraged the students to form study groups and create WeChat groups for online communication. Such practices would provide students with more opportunities for behavioral interaction. Additionally, after forming study groups, the students in each study group were required to develop a group contract to set shared learning goals and clarify rewards and sanctions, peer responsibilities, and obligations.

## 4. Experimental Design

### 4.1. Participants and Course Context

Eighty-one junior undergraduates taking an online course (digital learning resource design and development) at a Chinese university were chosen as samples for the experiment. These participants were from two disciplinary backgrounds (i.e., ideological and political education and international Chinese language education). These participants were already acquainted with each other beforehand and took other courses in the same semester; however, these courses were conducted online, and they did not meet face-to-face. The participants were randomly divided into two groups, with one labeled the control group (CG) (*N* = 43) and the other labeled the experimental group (EG) (*N* = 38). The teaching teams of three teachers in both the CG and EG were identical, with no prior exposure to online instruction. The participants ranged in age from 21 to 22 years; 10 were male, and 71 were female. All participants had prior online learning experience, successfully completed the course without any dropouts, and provided all necessary data.

The course was delivered completely online without face-to-face (F-T-F) contact. This course aimed to help students comprehend key concepts of digital learning and principles of digital learning resource development. When completing the course, the students were expected to be able to design and develop digital learning resources (e.g., PowerPoint slides and micro-lectures). The course lasted for ten weeks and included six learning modules. The course resources in the six modules included prerecorded online lectures and PowerPoints. All videos were recorded in advance, and the teacher appeared in the prerecorded online lectures. Moreover, each module contained one online forum, and the learning tasks included watching teachers’ podcasts and completing online test items. The students were encouraged to ask questions, express opinions and emotions, and exchange opinions with each other in the online forum activities of each module. The teacher closed the forum when the learning session of each module was finished. Finally, the students were required to submit their design work (i.e., PowerPoint slides and a micro-lecture) at the end of the online course.

### 4.2. Experimental Procedure

During the ten-week online course, the CG members received lecture-based instruction throughout the course. The EG attended online lectures, as did the CG for the first three weeks. From week 4 to week 9, the researchers complemented six activities (Table 1) that were designed under the guidance of the SP-based teaching strategy among the EG. The following paragraphs describe the process of the researchers’ implementation of the experiment. Table 2 shows an overview of the experimental procedure.

(1) During the exposure phase (weeks 1–3), the DG and EG members received lecture-based instruction, including viewing online course resources, completing course-related tasks, and participating in discussion forums. The purpose of the exposure phase was to examine whether the two groups would have significant differences in their social presence and social network characteristics before the intervention.
(2) In the pretest phase (week 3), the CG and EG members were required to complete a test to assess their foreknowledge about digital learning resource design and development, which was learned during the exposure phase. After the pretest, all participants completed an online questionnaire survey addressing their perceived social presence during the exposure phase.
(3) During the treatment phase (weeks 4–9), the CG members continued receiving lecture-based instruction in the online course, while the EG members were provided with the intervention to promote their social presence. In week 4, the EG students were asked to participate in learner identification, learning expectations and learning contracts activities. They provided personal information, expressed learning expectations, and signed learning contracts on the course platform. Additionally, they completed study groups and group contracts this week. Specifically, they signed learning contracts, formed study groups, developed group contracts, and published the results on the platform. The formation of study groups was not assigned by the teacher but was determined by the students according to their hobbies, learning needs, course goals, etc. Furthermore, from weeks 4 to 9, teachers encouraged students to utilize emotional cues in their online interactions, such as incorporating humor and emojis in public communications.
(4) During the posttest phase (week 10), the CG and EG members were requested to submit design works, including PowerPoint slides and a micro-lecture. In addition, both groups were invited to complete a questionnaire survey assessing their perceived social presence.

### 4.3. Instruments

To examine the effect of the SP-based teaching strategy on the participants’ social presence, a two-round questionnaire survey with a pretest and posttest and a coding schema on the participants’ social presence were conducted at week 3 and week 10, respectively. Because the course objectives focused on the development of students’ competencies in using information and communications technology in teaching, the participants’ academic performance was indicated by the scores of their design (i.e., PowerPoint slides and a micro-lecture). The following paragraphs provide detailed information about the instruments used to address these factors.

The instruments used to measure the participants’ social presence included the social presence scale [89] and the social presence coding schema [50]. The social presence scale indicated the participants’ perceived social presence with nine items in three dimensions, demonstrating a high reliability with a Cronbach’s Alpha of 0.923. This scale uses a five-point Likert scale (from 1 = strongly disagree to 5 = strongly agree) to rate the extent to which the participants agreed or disagreed with each statement, with reliability and validity tests performed by experts. The social presence coding schema instrument includes three categories and twelve indicators. The three categories were affective response, interactive response, and cohesive response. To ensure the reliability of the coding, two researchers independently screened the discussion postings and coded the data. Any disagreements were resolved through discussion.

Regarding the participants’ foreknowledge, the researchers administered a standardized test designed by the teaching staff. The test aimed to assess the participants’ knowledge of the principles of designing and developing digital learning resources. The participants’ academic performance was indicated by the results of their design work (i.e., a micro-lecture and PowerPoint slides) submitted at week 10. Two teachers independently marked the students’ final work at the end of the online course according to the grading criteria, including aesthetics, the correctness of content, and the rationality and richness of the technology used. Any disagreements were resolved through discussion.

Finally, the factor of the participants’ online interaction was addressed by the frequency of their participation in the online forums of all the learning modules.

### 4.4. Data Collection

The researchers obtained ethics approval from the bioethics committee of the university (Human Experiments of Zhejiang Normal University, the code is ZSRT2024201) and consent from the teaching staff and participants before conducting the research. A standard test was used to assess the participants’ foreknowledge in week 3. Both the EG and the CG students were required to complete the survey simultaneously on the platform, and the camera was turned on to supervise the process of the pretest. The participants’ academic performance at the end of the online course was collected from the results of their final studies (i.e., PowerPoint slides and micro-lectures) in week 10.

To collect the data on the participants’ perceived social presence, a pretest and a posttest of their social presence questionnaire were posted online, and the link of the questionnaire was sent to the EG and CG through WeChat groups. The EG and CG members were required to complete the questionnaire in week 3 and week 10, respectively.

In addition, to provide behavioral evidence, the researchers investigated the occurrence and frequency of social presence indicators from online discussion postings generated by the participants in six discussion forums.

Finally, we generated network sociograms of the EG and the CG during the exposure and treatment phases. We investigated the interaction data (e.g., questioning and responding between students) in six discussion forums. Specifically, the participants’ interaction data during the exposure phase were gathered from two online forums in learning modules 1–2. Their interaction data during the treatment phase were collected from four online forums in learning modules 3–6.

### 4.5. Data Analysis

A t test was conducted to examine the differences in students’ perceived social presence scores and foreknowledge between the EG and the CG. The level of significance was set to 95%. As the non-normal distribution of the data indicated the EG members’ academic performance, the Mann–Whitney U test was used to compare the difference in academic performance scores between the EG and the CG students.

The researchers coded students’ discussion postings in discussion forums according to the social presence coding schema developed by Rourke et al. (1999) [50] and used content analysis to examine the social presence participation of both the EG and the CG in three categories of social presence during online communication. The unit of analysis is a sentence or paragraph that conveys important thoughts or ideas. A total of 158 units were collected (see Table 3).

Additionally, Spearman’s correlation analysis was performed to examine the correlation between students’ academic performance and their perceived social presence scores.

Moreover, social network analysis could offer a better understanding of the relationships between participants and the characteristics of students’ online interactions. The interaction data were entered into UCINET6 for Windows [90], and we used social network analysis to analyze the network sociograms, social network characteristics, and cohesive subgroups of the EG and the CG. The sociograms showing the network structure are depicted with lines (representing relationships between people) and nodes (representing individual people) [91]. The variables in the social network analysis we examined included density, average distance, distance-based cohesion, and hybrid reciprocity. The density indicates the level of participants’ engagement in the network [92]. The cohesion measured through the geodesic distance between two participants reveals the degree of centralization of communication in the discussion forums [92]. Reciprocity indicates the pairwise relationship between two nodes (i.e., the ratio of mutual dyads to all possible dyads) [93]. Finally, researchers investigated the number of cohesive subgroups of the EG and CG through analysis of the cliques of the network. A cohesive subgroup is a “tightly knit” subset of actors in a social network [94]. A clique embodies a perfect cohesive subgroup where every two actors in the subgroup are directly connected to each other [95]. In this study, the maximum distance between two members in a subgroup was two, and the minimum number of members in a subgroup was four.

## 5. Results

### 5.1. Social Presence

The results of the t test showed that there was no statistically significant difference in students’ perceived social presence at the pretest. At the posttest, the EG members showed a significantly greater perceived social presence than the CG (Table 3).

Table 4 shows the behavioral evidence of participants’ online interactions in discussion forums, representing students’ contributions to the three categories of social presence from the exposure phase to the treatment phase. During the exposure phase, there was no significant difference in the total number of contributions to each category of social presence. The EG made two more analysis units than the CG, and the difference was mainly focused on the interactive response category. During the treatment phase, the gap in the total number of analysis units between the two groups became larger, with the EG having 45 more analysis units than the CG. Among the categories of increased social presence in the EG, affective response increased the most, followed by cohesive response and finally interactive response, whereas the CG increased only in the interactive response category.

### 5.2. Social Network Characteristics

**Figure 2:**
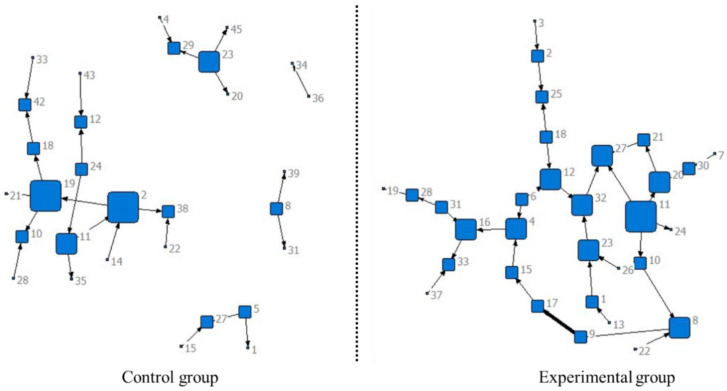
Network sociograms of the exposure phase.

**Figure 3:**
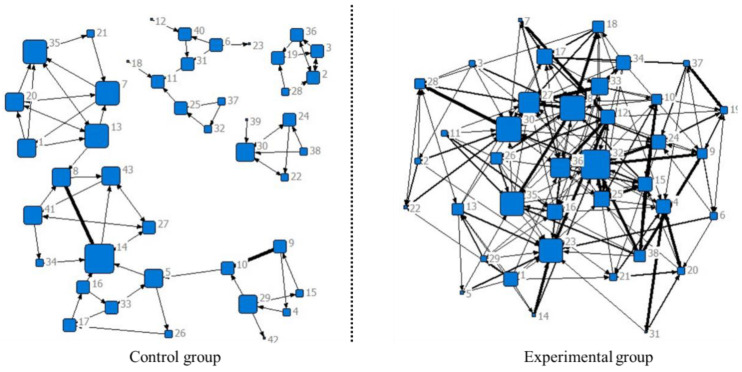
Network sociograms of the treatment phase.

Figure 2 and Figure 3 display the network sociograms of the EG and the CG during the exposure and treatment phases, respectively. The size of each node represents the degree of each member’s total interaction frequency with others. The arrows indicate the direction of the online interaction between the participants. The thickness of the lines indicates the total interaction frequency between two participants.

During the exposure phase, the researchers found twelve isolated nodes in the CG and seven isolated nodes in the EG. In addition, there were some connections, loose structures, and weak links in both the CG and the EG.

During the following phase of treatment, the isolated nodes in the CG and EG disappeared. The structure of the CG was still loose, while the structure of the EG was more concentrated. The number of connections between the group members in both the CG and EG increased, but there were more remarkable increases in connections between the group members in the EG compared to those in the CG. Regarding the strength of the links among the group members in the CG and EG, the EG demonstrated strengthened connections among its members, while there was no marked change in the CG from the exposure to the treatment phase.

The researchers identified more features of the network sociograms in Table 5. During the exposure phase, the density, average distance, and cohesion of the EG were slightly greater than those of the CG. Additionally, the reciprocity in both the EG and CG was zero.

During the treatment phase, the density and cohesion of the EG were greater than those of the CG, and the gap between the EG and CG became larger. In addition, despite the greater average distance traveled by the EG than by the CG in the exposure phase, the average distance traveled by the EG was lower than that traveled by the CG in the treatment phase. However, the reciprocity of the CG was slightly greater than that of the EG. Approximately 20% of the relationships unilaterally initiated by the EG and CG members were replied to by others and formed a dialog.

Finally, the researchers examined the number of cohesive subgroups of the EG and CG. There were no eligible subgroups in either the control or the experimental groups during the exposure phase.

During the treatment phase, the CG formed one subgroup. A total of 9.3% of the CG students appeared in the subgroups (Table 6). The EG members formed 11 subgroups, and 68.4% of all the EG students appeared in the subgroups (Table 6). Moreover, many members of the EG, such as members 9, 17, and 24, appeared in multiple subgroups at the same time, indicating that they actively interacted with others.

### 5.3. Academic Performance

The researchers found no significant difference between the two groups in terms of foreknowledge during the exposure phase (Table 7). Due to the non-normal distribution of the data indicating the EG members’ academic performance, we used the Mann–Whitney U test to investigate the effect of the SP-based teaching strategy on students’ academic performance at the end of the online course. As shown in Table 8, there was a significant difference between the two groups (*U* = 462.5, *z* = −3.366, *p* = 0.001), indicating that EG members were more capable of designing and developing digital learning resources than CG members were.

Pearson’s correlation analysis and Spearman’s correlation analysis were conducted to investigate the correlation between the participants’ academic performance and perceived social presence. The CG students’ academic performance was not significantly related to their perceived social presence. In contrast, there was a significant correlation between EG members’ academic performance and perceived social presence (Table 9).

## 6. Discussion and Implications

This study proposed an SP-based teaching strategy to support online teachers in designing learning activities from three dimensions of SP. Furthermore, the experimental results indicate that the intervention of the SP-based teaching strategy could improve students’ online learning in several aspects, such as perceiving significantly greater social presence, participating in more categories of social presence, achieving significantly better academic performance, and experiencing more frequent online interaction. This implies that teachers could enhance students’ social presence through modifications in their instructional design without necessarily relying on complex technological tools. The SP-based teaching strategy was shown to be effective in assisting online teachers in smoothly engaging in online teaching and enhancing students’ learning.

In terms of the students’ perceived social presence during the exposure phase, the researchers did not find any significant difference in the participants’ perceived social presence between the EG and CG. However, during the treatment phase, the EG members perceived greater social presence than those in the CG. This difference can be attributed to the SP-based teaching strategy intervention, which enhances students’ perceived social presence through online activities that promote co-presence, psychological involvement, and behavior engagement.

Additionally, before the intervention, the participants in the EG and CG mainly responded interactively to the course forums. Both the EG and CG members rarely posted affective and cohesive responses to the course forums. After receiving the intervention during the treatment phase, the EG members posted more interactive, affective, and cohesive responses to online discussion forums, while there were no such remarkable increases among the CG members. We designed emotional disclosure activities to promote students’ psychological involvement, which helps explain why the EG group exhibited more affective responses during the treatment phase. Moreover, emotional interaction has a positive effect on students’ learning engagement (e.g., interactive responses) [96,97]. On the other hand, we encouraged students to form study groups and use a mobile instant messaging-facilitated (MIM) app (i.e., WeChat) for online communication to enhance students’ behavioral engagement. Tang and Hew (2020) [98] examined to what extent social presence was presented in a MIM environment and compared it with that in a threaded discussion forum. They found that the MIM promoted greater online social presence among students than the asynchronous threaded forum. Therefore, the use of WeChat may encourage EG members to post more contributions to online study groups, and enriched online communication more extensively represents the three categories of social presence on platform forums.

Regarding the participants’ online interactions during the exposure phase, the social network analysis indicated that there was no significant difference in the network sociograms or social network characteristics between the EG and CG. However, during the treatment phase, compared with the CG, the EG displayed a more concentrated network sociogram, with more connections, stronger strength of links, greater density and cohesion, and lower average distance. Previous studies have also shown a positive relationship between students’ social presence and their online interactions [13,69,71]. As indicated by Tirado et al. (2015) [92], the cohesion index and the centralization index of the social network sociogram have a positive influence on students’ social presence. This finding implies that the intervention of the SP-based teaching strategy could promote students’ behavioral engagement, which might in turn improve their social presence. Importantly, this study revealed that the EG formed more subgroups than did the CG, and more EG members appeared in multiple subgroups at the same time during the treatment phase. This implies that the interaction among students in the EG became dense, which could facilitate students’ knowledge construction [99].

Regarding the students’ academic performance at the end of the online course, the EG members showed significantly better performance than the CG members, while there was no difference in students’ foreknowledge. Furthermore, the researchers found that the academic performance of the EG was significantly correlated with perceived social presence, while such a significant correlation was not identified for the CG. This finding indicates that the effect of social presence on EG students’ academic performance was stronger than that on CG students’ academic performance. Previous studies have identified a positive relationship between students’ social presence and academic performance [19,20,21]. This study further revealed that a positive relationship between students’ social presence and academic performance may exist only under high social presence conditions.

A few limitations were identified in the present study due to the restriction of sampling. First, the majority of the participants specialized in social and human science at a Chinese university. Therefore, the present findings cannot be generalized to a large student population in other subject areas. Future research should further examine the effect of the SP-based teaching strategy on students’ learning, with consideration of different demographic backgrounds (e.g., age, cultural background, and discipline background). Additionally, we did not further investigate how the three dimensions of social presence (i.e., co-presence, psychological involvement, and behavioral engagement) interact with each other during the intervention process to increase participants’ social presence and improve their academic performance. In the future, researchers can further investigate how each dimension of social presence could influence students’ positive online learning experiences and performance.

## 7. Conclusions

Improving students’ learning experience and performance has been identified as one of the most challenging tasks in online education. Due to insufficient support and training to prepare teachers for online teaching, online teachers rely heavily on lecture-based instruction, such as prerecorded lectures. They might consider lecture-based online instruction to be a practical teaching approach for delivering course content. However, they may overlook the important role of other factors (e.g., social and emotional support and peer interaction) on students’ learning experience and performance. Therefore, courses that are mainly delivered by lecture-based instruction and lack learning support are very likely to result in unsatisfactory learning experiences for the students. The contribution of the current study highlights the importance of social presence in online learning by proposing an SP-based teaching strategy and approving the effectiveness of this teaching strategy with empirical evidence. This study promotes the practical application of social presence in teaching.

Moreover, the results of this study further suggest that social presence can be improved even when complicated technological tools are not applied in online teaching. Specifically, instructors and course builders can develop some well-designed online activities with sound pedagogical consideration and can effectively improve students’ perceived social presence and participation in three categories of social presence. In addition, when designing training and professional development programs for online teaching, relevant institutions should not only help teachers master the necessary technological tools but also include a specific amount of content related to teaching theories and strategies. Ensuring a seamless integration of theory and technology is essential for effective online instruction.

## Figures and Tables

**Table 1:** Activities designed under the guidance of the SP-based teaching strategy.

Dimension	Activity Name	Description
Co-presence	Learner identification	Filling in personal information on the course platform (e.g., name, grade, age, specialty, and hobby).
Psychological involvement	Learning expectations	Expressing their online learning needs and expectations.
	Learning contracts	Following the rules for rewards and punishments during online learning.
	Emotional disclosure	Providing emotional cues during online communication (e.g., humor and emoticons in public).
Behavioral engagement	Study groups	Forming study groups and creating WeChat groups for online communication.
	Group contracts	Setting shared learning goals and clarifying reward and punishment, peer responsibilities, and obligations.

**Table 2:** Overview of the procedure of the experimental study.

Phase	Duration
(1) Exposure phase The EG and CG participated in the lecture-based online learning activities in learning modules 1–2.	Week 1–3
(2) Pretest phase The EG and CG were assessed on their perceived social presence and foreknowledge.	40 min in Week 3
(3) Treatment phase The CG would receive the lecture-based instruction in learning modules 3–6. The EG would receive the lecture-based online learning activities and intervention in learning modules 3–6.	Week 4–9
(4) Posttest phase The EG and CG were assessed on their perceived social presence and academic performance.	70 min in week 10
Total time	Ten weeks

**Table 3:** A comparison of student’s perceived social presence between the control and experimental groups pre- and posttests.

	Group	N	Mean	SD	*t*	*d*
Pretest	CG	43	16.42	6.24	1.202	0.27
	EG	38	14.74	6.34		
Posttest	CG	43	22.21	5.10	−7.486 ***	1.68
	EG	38	31.18	5.69		

Note. CG = control group; EG = experimental group; N = number; SD = standard deviation; *t* = T-value; *d* = Cohen’s effect size; *** *p* < 0.001.

**Table 4:** Units of analysis.

Phase	Group	Category			
		Affective Response	Interactive Response	Cohesive Response	Total
Exposure	EG	0	8	1	9
	CG	1	5	1	7
Treatment	EG	25	14	15	54
	CG	1	7	1	9

Note. CG = control group; EG = experimental group.

**Table 5:** Structure of network sociograms.

Group	Phase	Density ^a^	Average Distance	Distance-Based Cohesion ^b^	Hybrid Reciprocity ^c^
CG	Exposure	0.0138	1.940	0.019	0.0000
	Treatment	0.0437	2.957	0.080	0.2222
EG	Exposure	0.0235	2.540	0.036	0.0000
	Treatment	0.2020	2.605	0.444	0.2152

Note. CG = control group; EG = experimental group; ^a^ Range 0–1, higher values indicate a greater connection; ^b^ Range 0–1, higher values indicate greater cohesiveness; ^c^ Range 0–1, higher values indicate a greater proportion of reciprocity.

**Table 6:** Subgroups of the CG in the treatment phase.

Group	Members
CG	14, 27, 41, 43
EG	9, 10, 15, 25, 32, 38
	9, 15, 24, 32, 38
	9, 19, 24, 32, 38
	6, 9, 19, 24
	4, 20, 21, 31
	7, 8, 17, 36
	7, 17, 30, 36
	7, 12, 17, 36
	8, 17, 18, 33
	8, 27, 33, 34
	13, 22, 28, 30

Note. CG = control group; EG = experimental group.

**Table 7:** A comparison of student’s foreknowledge.

Group	N	Mean	SD	*t*	*d*
CG	43	85.49	4.50	−1.088	0.24
EG	38	86.74	5.81		

Note. CG = control group; EG = experimental group; N = number; SD = standard deviation; *t* = T-value; *d* = Cohen’s effect size.

**Table 8:** A comparison of students’ academic performance.

Group	N	Mean	SD	*p*
CG	43	88.29	2.83	0.001
EG	38	89.86	1.96	

Note. CG = control group; EG = experimental group; N = number; SD = standard deviation; *p* = *p*-value.

**Table 9:** A bivariate correlation coefficient of students’ perceived social presence and academic performance.

		SD	Correlation Coefficient
CG	Academic performance	2.83	0.25
EG	Academic performance	1.96	0.41 *

Note. CG = control group; EG = experimental group; SD = standard deviation; * Correlation is significant at the 0.05 level (2-tailed).

## Data Availability

The original contributions presented in the study are included in the article, further inquiries can be directed to the corresponding author.

## Abbreviations

Social presence	SP
Experimental group	EG
Control group	CG
Face-to-face	F-T-F
Emergency remote teaching	ERT
Community of inquiry	COI
Mobile instant messaging-facilitated	MIM
